# Differentiating T1a–T1b from T2 in gastric cancer lesions with three different measurement approaches based on contrast-enhanced T1W imaging at 3.0 T

**DOI:** 10.1186/s12880-021-00672-7

**Published:** 2021-09-28

**Authors:** Yuan Yuan, Shengnan Ren, Tiegong Wang, Fu Shen, Qiang Hao, Jianping Lu

**Affiliations:** 1 Department of Radiology, Changhai Hospital of Shanghai, No.168, Shanghai, China; 2 Department of Nuclear Medicine, Shanghai Fourth People’s Hospital, Shanghai, China

**Keywords:** Gastric cancer, Magnetic resonance imaging, T staging, Volume, Thickness

## Abstract

**Background:**

To explore the diagnostic value of three different measurement approaches in differentiating T1a–T1b from T2 gastric cancer (GC) lesions.

**Methods:**

A total of 95 consecutive patients with T1a–T2 stage of GC who performed preoperative MRI were retrospectively enrolled between January 2017 and November 2020. The parameters MRI T stage (subjective evaluation), thickness, maximum area and volume of the lesions were evaluated by two radiologists. Specific indicators including AUC, optimal cutoff, sensitivity, specificity, accuracy, positive likelihood ratio (PLR), negative likelihood ratio (NLR), positive predictive value (PPV) and negative predictive value (NPV) of MRI T stage, thickness, maximum area and volume for differentiating T1a–T1b from T2 stage lesions were calculated. The ROC curves were compared by the Delong test. Decision curve analysis (DCA) was used to evaluate the clinical benefit.

**Results:**

The ROC curves for thickness (AUC = 0.926), maximum area (AUC = 0.902) and volume (AUC = 0.897) were all significantly better than those of the MRI T stage (AUC = 0.807) in differentiating T1a–T1b from T2 lesions, with *p* values of 0.004, 0.034 and 0.041, respectively. The values corresponding to the thickness (including AUC, sensitivity, specificity, accuracy, PPV, NPV, PLR and NLR) were all higher than those corresponding to the MRI T stage, maximum area and volume. The DCA curves indicated that the parameter thickness could provide the highest clinical benefit if the threshold probability was above 35%.

**Conclusions:**

Thickness may provide an efficient approach to rapidly distinguish T1a–T1b from T2 stage GC lesions.

## Background

Gastric cancer (GC) is the fifth most common cancer and has caused more than 780,000 deaths worldwide in 2018 [1]. Early GC usually requires less intense treatment and exhibits better prognosis compared with advanced GCs. Patients with Tis and T1a stage lesions can receive minimal invasive treatment with endoscopic resection, whereas patients with T1b lesions can be treated by a direct surgery. In contrast to T1b lesions, patients presenting with T2 stage GC lesions prefer a perioperative chemotherapy to increase the probability for curative resection and to improve the survival rate according to the latest National Comprehensive Cancer Network (NCCN) Guidelines [2–4].

CT is the most conventional noninvasive preoperative assessment method of GC, but it is reported that CT is not capable of measuring the depth of early GC or the rest normal tissue in deeper layer of gastric wall and most of the early GC were not able to detect due to its poor soft tissue resolution [5–7]. However, magnetic resonance imaging (MRI) is a promising technique with high soft tissue resolution used in the evaluation of GC [8]. Currently, no worldwide criteria have been defined with regard to T1 stage lesions in MRI [8, 9]. The majority of the previous studies described visible lesions that were enhanced in tissues that did not exceed the submucosal layer as T1 stage based on MRI [10–14]. A limited number of studies described focal thickening of the inner layer of the gastric wall with slight enhancement as T1 stage tumors in MRI [15, 16]. The overall accuracy of diagnosing T1 stage by MRI ranged from 50 to 100% using a sample size of 1 to 12 [10–12, 14–17]. These findings could be severely biased. Moreover, there was subjectivity in applying the aforementioned criteria and the observer agreement in evaluating the T1 and T2 stages with contrast-enhanced T1W imaging (CE-T1WI) was moderate in some studies [13, 16].

Other studies evaluated the diagnostic value of CT volumetry in T staging of GC tumors and demonstrated the ability of predicting the T1 stage, with a sample size of 19 and 13 patients, respectively and with an accuracy of 80.7% and 95% in differentiating T1 stage from other higher stages, respectively [18, 19]. Furthermore, the thickness of GC in untreated patients was relatively stable which was not affected by the degree of gastric filling [5]. These results indicated high potential of this method for evaluating T staging in GC. To achieve optimal management for patient benefit, the accurate preoperative distinction of T1a–T1b from T2 stages is essential. To the best of our knowledge, the application of this diagnostic method has not been examined before, with the exception of the parameter tumor volume. Therefore, a precise and reproducible method of distinguishing T1 from T2 stage in GC is vital.

The purpose of the present study was to investigate the diagnostic value of thickness in differentiating T1a–T1b from T2 GC lesions in contrast-enhanced MRI (CE-MRI).

## Methods

The present study was approved by our institutional review board and informed consent was waived for every patient.

### Patients

**Fig. 1:**
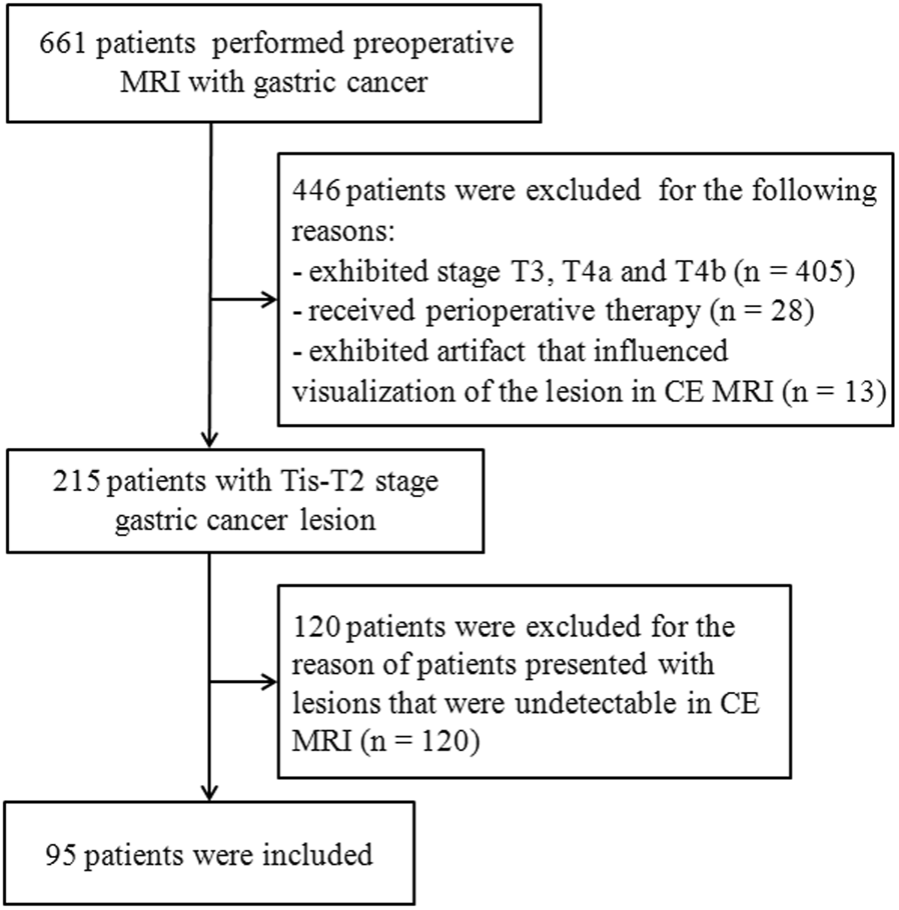
Flowchart shows the inclusion and exclusion criteria

We retrospectively reviewed the PACS of 661 consecutive patients with GC who underwent preoperative stomach MRI from January 2017 to November 2020. The present study included patients with pathologically confirmed primary GC who underwent surgery within 1 week following MRI examination and received pathological diagnosis in the end. Patients were excluded if the pathological T stages were T3, T4a or T4b and in case of preoperative therapy, such as chemotherapy, radiotherapy and endoscopic resection. Patients were excluded if they exhibited poor image quality resulting in poor visualization of lesions and in case of lesions that were undetectable in CE-MRI (Fig. 1). Two radiologists (Y.Y. and T.W. both with 10 years of experience in abdominal radiology, respectively) determined the detection efficacy in consensus, according to the criterion of a definite visualization of the GC lesion. Sex, age, CEA level, CA199 level, CA724 level, BMI index of all included patients were recorded.

### MRI acquisition

Each patient was fasted for at least 5 h and warm water (500 ml) was administered to dilate the stomach before the MRI examination. No drugs were injected to inhibit gastrointestinal peristalsis. All MRI examinations were performed on one scanner (3.0 T MAGNETOM Skyra, Siemens Healthcare, Germany). 18-channel phased-array body and integrated spine coils were applied to obtain the MRI signal. The sequences were uniformed as a settled image set as follows: axial T2WI, three dimensional T1WI volume interpolated body examination (3D T1WI VIBE) and three phases of CE-T1WI (arterial, venous and delayed phases). The parameters for T2WI were as follows: TR, 4560 ms; TE, 79 ms; FOV, 380 * 380 mm^2^; matrix, 320 * 320; flip angle 140°; slice thickness, 6 mm; gap, 1.2 mm; fat saturation, yes; acquisition time 3–4 min. The following parameters were used for CE-T1WI: TR, 3.46 ms; TE, 1.32 ms; FOV, 308 * 380 mm^2^; matrix, 195 * 320; flip angle 12°; slice thickness, 3 mm; gap, 0 mm; fat saturation, yes; acquisition time, 14 s. The CE-T1W images were obtained at 30, 60, 90 s following contrast injection, which consisted of 0.2 ml/kg body weight Gd-DTPA (Beilu, Beijing, China) delivered using an automatic power injector (Medrad Spectris Solaris EP MR Injector System, PA, USA) at 2 ml/s followed by a 20 ml saline flush at the same rate.

### Image analysis

All images were transferred to GE PACS RA1000 for further analysis. Two abdominal radiologists (Y.Y. and T.W. both with 10 years of experience in abdominal radiology, respectively) who were aware of the existence but not aware of any other clinical information of all GC lesions were selected for independent evaluation of the location (three categories of fundus, body and antrum), the MRI T stage (subjective evaluation of ≤ T1 or ≥ T2 with combination of T2W and CE-T1WI). The researchers reached a consensus when encountering inconsistent diagnosis. The MRI T stage was determined according to the AJCC 8th edition GC staging. The MRI T stage criteria were as follows: enhanced tissues not exceeding the submucosal layer were termed T1a–T1b stage, whereas enhanced tissues exceeding the submucosal layer were termed T2 stage [10, 20].

**Fig. 2:**
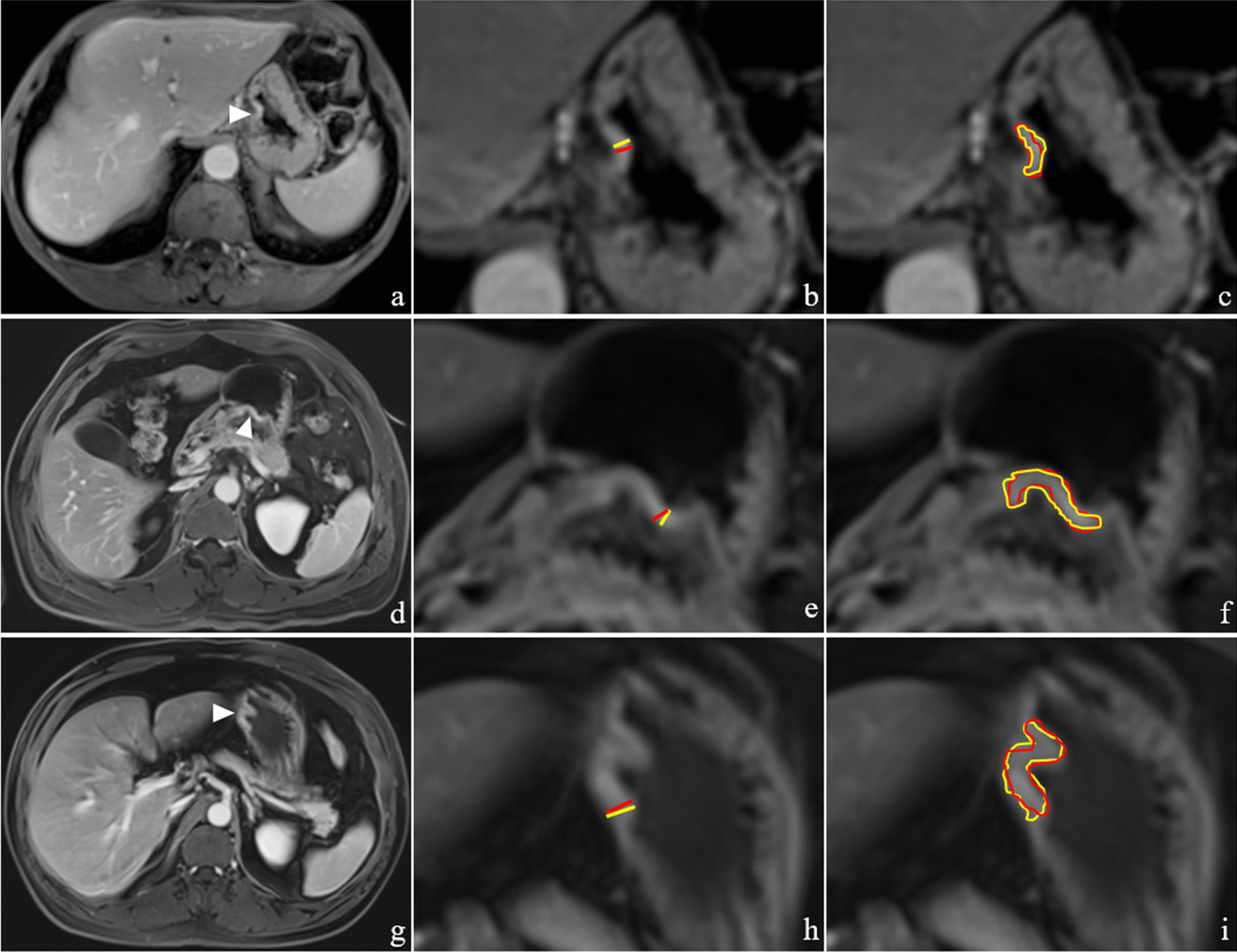
Example images for the measurements of GC lesions. **a**–**c** Images were from a patient with gastric fundus cancer (white arrowhead), confirming as pT1a (**a**), with thickness measurements of 2.4 and 2.7 mm (**b**), maximum area measurements of 40.7 and 36.8 mm^2^ (**c**). **d**–**f** Images were from a patient with gastric antrum cancer (white arrowhead), confirming as pT1b (**d**), with thickness measurements of 2.8 and 3.4 mm (**e**), maximum area measurements of 146.8 and 153.6 mm^2^ (**f**). **g**–**i** Images were from a patient with gastric body cancer (white arrowhead), confirming as pT2 (**g**), with thickness measurements of 5.2 and 5.7 mm (**h**), maximum area measurements of 170.4 and 177.9 mm^2^ (**i**). Red line and ROI indicating radiologist 1. Yellow line and ROI indicating radiologist 2

The parameters thickness, maximum area and volume were evaluated based on CE-T1WI (venous phase). All the lesions were moved to the center of the screen and zoomed in two times, so that two radiologists could independently measure the lesions in order to achieve more precise measurements within one week. Thickness was measured at the thickest part of the maximum area which presented as abnormal high signal intensity perpendicular to the gastric wall direction. The regions of interest (ROIs) were drawn along the edge of the lesion according to abnormal signal intensity compared with the adjacent normal gastric wall on each slice within one week. The maximum area was selected from the ROIs (Fig. 2). The volume was estimated by the following formula: volume = sum of area of ROIs × slice thickness. The average value of thickness, maximum area and volume was provided by two radiologists for final analysis.

### Pathological evaluation

All specimens were pathologically confirmed as GC. The location and T stage were assessed. The T stage was determined according to the American Joint Committee on Cancer (AJCC, 8th edition). The tumor invasion of the lamina propria or muscularis mucosae was considered T1a, whereas the tumor invasion of the submucosa was considered T1b and the tumor invasion of the muscularis propria was considered T2 [20]. T1a and T1b were grouped together, whereas T2 was an independent group.

### Statistical analysis

The Kolmogorov–Smirnov statistical test was performed to assess the normality for all continuous variables. The sample t test or Mann–Whitney U test were performed to compare continuous variables. The Chi-square test was performed to compare qualitative data. The parameters AUC, optimal cutoff (if required), sensitivity, specificity, accuracy, positive likelihood ratio (PLR), negative likelihood ratio (NLR), positive predictive value (PPV), negative predicative value (NPV) of MRI T stage, thickness, maximum area and volume for differentiating T1a–T1b from T2 lesions were calculated. The Delong test was performed to compare the ROC curves. Kappa or weighted Kappa coefficient was estimated to assess the interobserver agreement in the qualitative data. A kappa value > 0.8 indicated excellent agreement, whereas those from 0.6 to 0.8, 0.4 to 0.6, 0.2 to 0.4 and 0.0 to 0.2 indicated substantial, moderate, fair and slight agreement, respectively. A kappa value < 0.0 indicated poor agreement [21]. The intraclass correlation coefficient (ICC) was performed for continuous variables (ICC = 0 to 0.49, poor agreement; ICC = 0.50 to 0.75, moderate agreement; ICC = 0.76 to 0.90, good agreement; ICC = 0.91 to 1.00, excellent agreement) [22]. The Bland–Altman analysis was performed for continuous variables to evaluate the interobserver agreements of two radiologists. *P* < 0.05 was considered to indicate significant differences. The MedCalc software (version 19.6.1) and the SPSS software (version 20.0) was used to calculate the weighted Kappa coefficient, perform ROCs and other analysis. The decision curve analysis (DCA) was performed with R (R version 3.3.3).

## Results

### Patients

Among the 661 patients, 566 patients were excluded since they exhibited stage T3, T4a and T4b (*n* = 405) lesions. A part of these patients received preoperative therapy (*n* = 28) and some of them exhibited an artifact that influenced visualization of the lesion in CE-MRI (*n* = 13). A total of 120 patients presented with lesions that were undetectable in CE-MRI (*n* = 120). Finally, 95 patients were included.

### Clinical and pathological characteristics

All 95 patients (65 males, 30 females, mean age 62 ± 12 [standard deviation] years, range from 26 to 84 years) were treated with surgery and exhibited pathological data. A total of 8 lesions were present of T1a stage, whereas 31 lesions of T1b stage and 56 lesions of T2 stage were also noted. The mean BMI index of all patients was 23.1 ± 3.4 [standard deviation] kg/m^2^, range from 16.5 to 32.4 kg/m^2^. The data for the tumor markers CEA, CA199 and CA724 were missing in two patients. A total of 18 patients were present whose CEA levels were > 5 ng/ml, whereas 75 patients exhibited CEA levels ≤ 5 ng/ml. A total of 5 patients were selected whose CA199 level was > 37 U/ml, whereas 88 patients exhibited CA199 levels ≤ 37 U/ml. There were 9 patients with CA724 levels > 9.8 U/ml and 84 patients with CA199 levels ≤ 9.8 U/ml. A total of 27 lesions were located at fundus, whereas 17 lesions were located at the body and 51 lesions at the antrum. All clinical and pathological characteristics of the T1a–T1b and T2 GC lesions are shown in Table 1.

**Table 1:** Clinical characteristics in patients with T1a-T1b and T2 stage GC lesions

Variable	T1a–T1b (*n* = 39)	T2 (*n* = 56)	*P* value
Age (y)	61 ± 13	63 ± 12	0.396
*Sex*			
Male	25	40	0.450
Female	14	16	
BMI index (kg/m^2^)	23.2 ± 3.1	23.1 ± 3.6	0.880
*CEA level*			0.175
≤ 5 ng/ml	34	41	
> 5 ng/ml	5	13	
Missing		2	
*CA199 level*			0.137
≤ 37 U/ml	39	49	
> 37 U/ml	0	5	
Missing		2	
*CA724 level*			0.106
≤ 9.8 U/ml	38	46	
> 9.8 U/ml	1	8	
Missing		2	
*Tumor location*			0.850
Fundus	11	16	
Body	8	9	
Antrum	20	31	

### Measurements

The median thickness of all patients was 5.650 mm [IQR, 4.400], ranging from 1.800 to 18.900 mm. The median maximum area of all patients was 149.500 mm^2^ [IQR, 255.250], ranging from 10.600 to 1788.100 mm^2^. The median volume of all patients was 1680.600 mm^3^ [IQR, 3437.250], ranging from 72.800 to 50,638.500 mm^3^. The median thickness of T1a–T1b stage lesions (3.250 mm, [IQR, 1.150]) was significantly lower than that of the T2 stage (6.650 mm, [IQR, 5.300]) (*p* < 0.0001) lesions. The median maximum area of the T1a-T1b stage (55.700mm^2^, [IQR, 72.600]) lesions was significantly lower than that of the T2 stage (257.350mm^2^, [IQR, 367.350]) (*p* < 0.0001) lesions. The median volume of the T1a–T1b stage (471.600 mm^3^, [IQR, 806.100]) lesions was significantly lower than that of the T2 stage (3300.270 mm^3^, [IQR, 6387.980]) (*p* < 0.0001) lesions. The MRI measurements in patients with T1a–T1b and T2 GC lesions are shown in Table 2.

**Table 2:** CE MRI measurements in patients with T1a–T1b and T2 stage GC lesions

Measurement	T1a–T1b (*n* = 39)	T2 (*n* = 56)	*P* value
Median thickness (mm)	3.250 (IQR, 1.150)	6.650 (IQR, 5.300)	< 0.0001
(range, mm)	1.800–7.000	2.650–18.850	
Median maximum area (mm^2^)	55.700 (IQR, 72.600)	257.350 (IQR, 367.350)	< 0.0001
(range, mm^2^)	10.600–579.700	676.950–1788.050	
Median volume (mm^3^)	471.600 (IQR, 806.100)	3300.270 (IQR, 6387.980)	< 0.0001
(range, mm^3^)	72.750–6874.200	698.000–50,639.000	

### Observer agreement

**Fig. 3:**
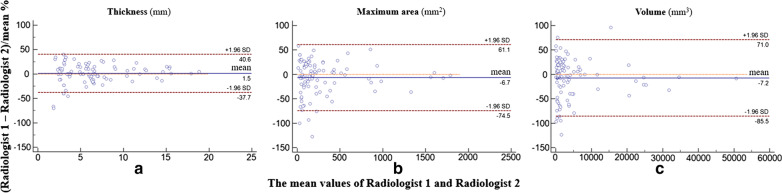
The Bland–Altman analysis in thickness, maximum area and volume of two radiologists. Solid line indicated the mean difference between two radiologists, dashed lines indicated the limits of the agreements (LoA, 1.96 standard deviations of the mean difference). 4.2% (4/95), 4.2% (4/95) and 6.3% (6/95) of all cases were outside of LoA in the measurement of the thickness (**a**), maximum area (**b**) and volume (**c**) respectively, indicating good consistency of all indexes measurements

The interobserver agreements between two radiologists are shown in Table 3. The consistency of the lesion location between the MRI and the pathology results was excellent (Kappa = 1.000, 95% CI: 1.000, 1.000). The interobserver agreements for the MRI T stage between the two radiologists was excellent (Kappa = 0.832, 95% CI 0.720, 0.943). The interobserver agreements for thickness, maximum area and volume were all excellent, with ICCs of 0.970 (95% CI 0.955, 0.980), 0.966 (95% CI 0.949, 0.977) and 0.958 (95% CI 0.938, 0.972), respectively. The Bland–Altman analysis in thickness, maximum area and volume of two radiologists is shown in Fig. 3.

**Table 3:** Interobserver agreements between two radiologists

Variable	Agreement (95%CI)
MRI T stage†	0.832 (0.720, 0.943)
Thickness*	0.970 ( 0.955, 0.980)
Maximum area*	0.966 (0.949, 0.977)
Volume*	0.958 (0.938, 0.972)

^†^kappa value

^*^Intraclass correlation coefficient

### Diagnostic performance for differentiating T1a-T1b from T2 lesions

**Fig. 4:**
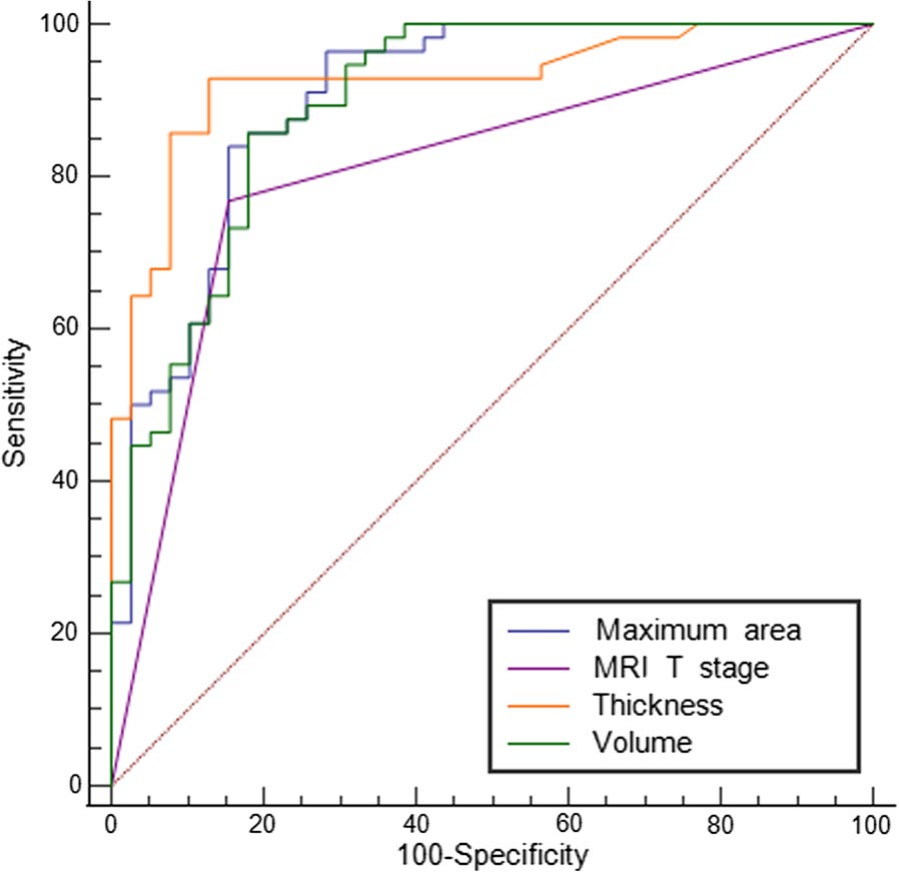
The ROC curves of MRI T stage, thickness, maximum area and volume for differentiating T1a–T1b from T2 stage GC lesions

**Fig. 5:**
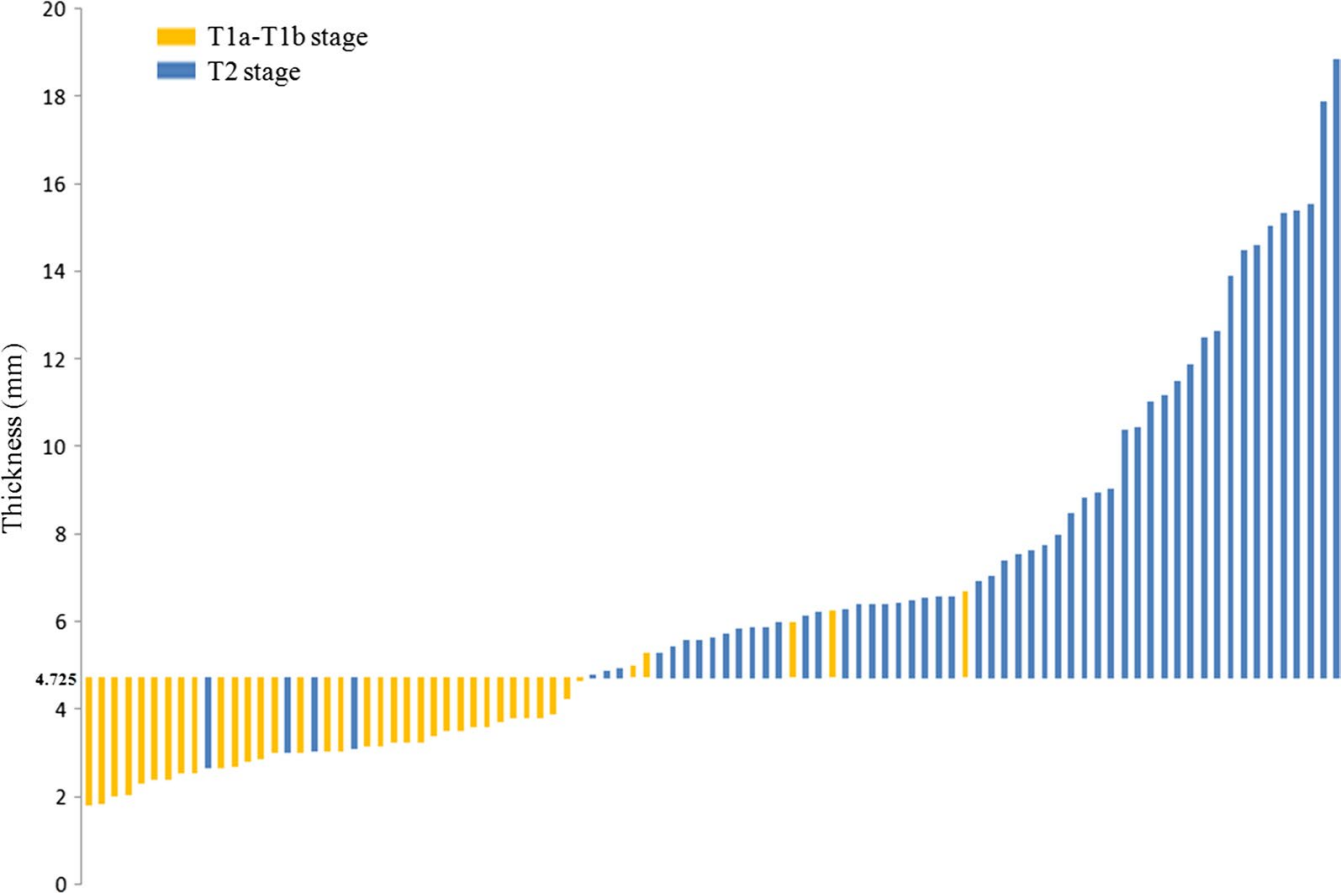
The thickness values for each lesion. The blue bars indicated the thickness value for T2 stages and the orange bars indicated the thickness value for T1a–T1b stages. The optimal cut off value for thickness was 4.725 mm

**Fig. 6:**
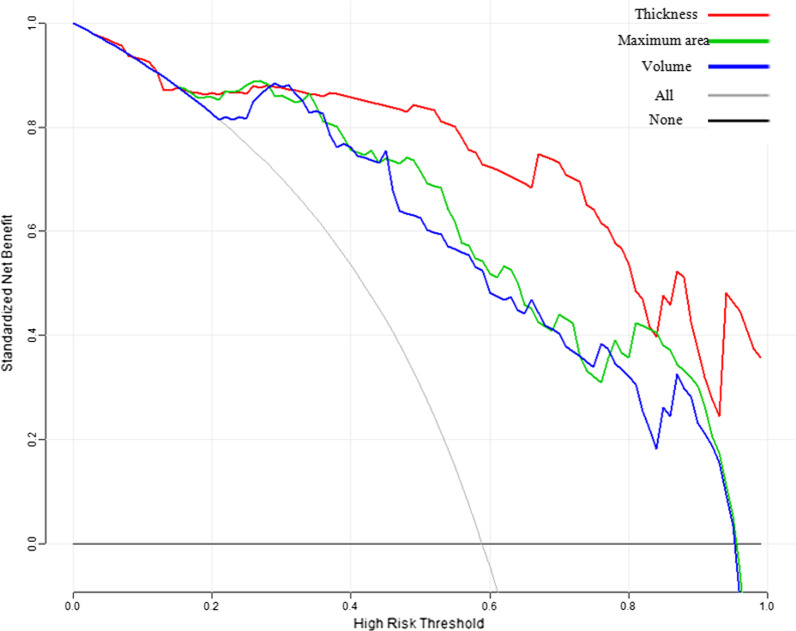
Decision curve analysis (DCA) for thickness, maximum area and volume in differentiating T1a–T1b from T2 stage GC lesions

The comparison of the ROC curves (Fig. 4) indicated that the parameters thickness, maximum area and volume were all significantly higher than those at the MRI T stage in differentiating T1a–T1b from T2 lesions, with AUC values of 0.926 compared to 0.807 (*p* = 0.004), 0.902 compared to 0.807 (*p* = 0.033) and 0.897 compared to 0.807 (*p* = 0.041), respectively. No significant differences were noted with regard to the three ROC curves for the parameters thickness, maximum area and volume (thickness compared to maximum area, *p* = 0.383, thickness compared to volume, *p* = 0.315, maximum area compared to volume, *p* = 0.315). The optimal cut off values for thickness, maximum area and volume were 4.725 mm (Fig. 5), 132.625 mm^2^ and 1343.850 mm^3^, respectively. The values of the parameters sensitivity, specificity, accuracy, PPV and NPV in thickness were all higher than those in MRI T stage, maximum area and volume. The value of PLR in thickness was distinctly higher than that in the MRI T stage, maximum area and volume (12.205 compared to 3.645, 5.265 and 5.744). The NLR for the parameter thickness was lower than that noted for the parameters MRI T stage, maximum area and volume (0.14 versus 0.20, 0.183 and 0.209). The detailed data are shown in Table 4. The DCA curves indicated that thickness provided the most clinical benefit when the threshold probability was higher than 35% (Fig. 6).

**Table 4:** Indicators of MRI T stage, thickness, maximum area and volume for differentiating T1a-T1b from T2 stage GC lesions

	MRI T stage	Thickness (mm)	Maximum area (mm^2^)	Volume (mm^3^)
AUC	0.807 (0.713,0.881)	0.926 (0.853,0.969)*	0.902 (0.823,0.954)*	0.897 (0.818,0.950)*
Optimal cut off	NA	4.725	132.625	1343.850
Sensitivity	0.846 (33/39)	0.872 (34/39)	0.846 (33/39)	0.821 (32/39)
Specificity	0.768 (43/56)	0.929 (52/56)	0.839 (47/56)	0.857 (48/56)
Accuracy	0.800 (76/95)	0.905 (86/95)	0.842 (80/95)	0.842 (80/95)
PPV(%)	0.717 (33/46)	0.895 (34/38)	0.786 (33/42)	0.800 (32/40)
NPV(%)	0.878 (43/49)	0.912 (52/57)	0.887(47/53)	0.873 (48/55)
PLR	3.645	12.205	5.265	5.744
NLR	0.200	0.138	0.183	0.209
*P* value*	NA	0.004	0.034	0.041

^*^Compared to MRI T stage, respectively by the Delong test

## Discussion

The present study indicated that the CE-T1WI values of the variables thickness, maximum area and volume were significantly higher than those of the MRI T stage in differentiating T1a–T1b from T2 stage GC lesions, with AUCs of 0.926 compared to 0.807 (*p* = 0.004), 0.902 compared to 0.807 (*p* = 0.034) and 0.897 compared to 0.807 (*p* = 0.041), respectively.

One meta-analysis demonstrated that MRI is a good diagnostic technique for preoperative T staging of GC. The detectability of GC is strongly influenced by tumor size, T-stage, histologic subtype and enhancement pattern of the gastric wall [23], thus the detectability of T1 stage of GC in MRI is poor, the reported pooled sensitivity of diagnosing T1 stage GC with MRI was 66% [23]. In one study, both anatomical MRI and diffusion-weighted imaging (DWI) were unable to locate the area of pathological tissue in all patients with pT1 tumors [24]. Another study reported detection in 16.3% (7/43) of pT1 tumors by anatomical MRI and 20.9% (9/43) by combined anatomical MRI and DWI [25]. Due to the low sensitivity for detecting early GC (23), a limited number of studies have been reported that examined the ability to differentiate T1a-T1b from T2 GC lesions in MRI. Liu S et al. reported that the accuracy of differentiating Tis-T1 from T2–T4 in CE-MRI was 96.1% (49/51). However, the study only included 20 cases of Tis-T2 GC lesions and mixed cases with T3 and T4 lesions. These factors may result in imprecise evaluation in differentiating T1 from T2 [16] lesions. To achieve optimal management for patient benefit, the accurate preoperative distinction of T1a–T1b from T2 stage is particularly important. The present study included 95 lesions of the T1a–T2 stage and revealed that the indices AUC, sensitivity, specificity and accuracy for differentiating T1a–T1b from T2 lesions in MRI T stage were 0.807, 0.846, 0.768 and 0.800, respectively.

Two previous studies [18, 19] evaluated the CT volumetry correlation with T stage in GC and demonstrated that CT volumetry was capable of distinguishing T1 stage from higher T stages in GC, which was consistent with our findings. The accuracies of differentiating T1 stage from higher T stages of the two previous studies were 95% and 80.7%, respectively [18, 19]. The optimal cut off values of CT volumetry for differentiating T1 from higher stages derived from the two studies were 19.4 and 8.2 cm^3^ [18, 19], respectively, which were both distinctly higher than the cutoff value noted in the present study (1.34 cm^3^). This inconsistency may be caused by the predominance of T4 stages in the studies reported previously or by the different methods used. However, the diagnostic value of thickness and maximum area for differentiating T1a–T1b from T2 GC lesions in MRI has not been previously investigated in the literature. According to our findings, the parameters thickness, maximum area and volume, based on CE-T1WI, were all significantly higher than those noted in the MRI T stage in differentiating T1a–T1b from T2 lesions. This finding is consistent with previous findings reporting that CT volumetry was significantly better than CT T-staging in predicting ≥ T2 [18]. The key point of differentiating T1a–T1b from T2 lesions in the MRI T stage is based on whether the lesion exceeds the submucosal layer. Ex vivo MR imaging studies demonstrated that this method can clearly depict the gastric wall layers [26–28]. However, in clinical practice, it was not possible to clearly distinguish each layer of the gastric wall, including lamina propria, muscularis mucosae, submucosa, muscularis propria, subserosal connective tissue and serosa. Even though we could identify the muscularis propria from time to time, we were usually not able to precisely evaluate the association between lesion and the muscularis propria due to current clinical resolution limits. The overall accuracy of subjective evaluation of MRI T stage ranged from 64 to 88% [9]. Summary receiver operating characteristic (SROC) of MRI to diagnose T1 stage showed an AUC of 0.6737 [23]. In addition, the interobserver agreement of MRI in T staging of GC is inconsistent in the literature, ranging from 0.578 to 0.970 [13, 15, 16]. Therefore, the subjective evaluation of MRI T stage may lead to unstable results. However, the measurements of thickness and volume are considered stable and high reproducible according to our findings and literature [5, 18, 19]. Thus we can focus on the measurement based on the abnormal signal intensity without subjectively evaluating the status of muscularis propria. The variables thickness, maximum area and volume demonstrated excellent observer agreement. These measurements may be used for clinical practice in the future. Among these three approaches, thickness measurement was the most efficient, volume measurement was the most time consuming.

No significant differences were noted between the three ROC curves corresponding to thickness, maximum area and volume. However, all the indicators (containing sensitivity, specificity, accuracy, PLR, NLR, PPV, NPV) of thickness examined, exhibited the highest efficacy among these three approaches. PLR (value = 12.21) is a comprehensive indicator that exhibits more meaningful diagnostic value compared to other indicators when the value is higher than 10. According to the DCA curves, thickness may provide the best clinical benefit for differentiating T1a–T1b from T2 lesions compared to the other two indices (maximum area and volume) when the threshold probability is above 35%.

The present study exhibited certain limitations. Firstly, it was a retrospective study with inevitable selection bias and the sample size was not large. Secondly, we only used CE-T1WI for evaluation and the values of other sequences, such as T2WI [29] and DWI [30] require further investigation. Thirdly, this was a single-center research study and additional studies in multi-centers were warranted to further confirm our findings.

## Conclusions

In conclusion, all the three approaches, namely thickness, maximum area and volume that were based on CE-T1WI provided better diagnostic performance than subjective evaluation of MRI T stage in differentiating T1a–T1b from T2 GC lesions. Thickness may provide the most efficient approach and gain the best clinical benefit among these three measurement approaches.

## Data Availability

The datasets used and/or analyzed during the current study are available from the corresponding author on reasonable request.

## Abbreviations

GC: Gastric cancer
MRI: Magnetic resonance imaging
ROC: Receiver operating characteristic
SROC: Summary receiver operating characteristic
AUC: Area under the curve
T1WI: T1-weighted imaging
CE: Contrast-enhanced
T2WI: T2-weighted imaging
DWI: Diffusion-weighted imaging
NCCN: National comprehensive cancer network
CT: Computed tomography
ROI: Region of interest
AJCC: American Joint Committee on Cancer
ICC: Intraclass correlation coefficient
PLR: Positive likelihood ratio
NLR: Negative likelihood ratio
PPV: Positive predictive value
NPV: Negative predicative value
DCA: Decision curve analysis
IQR: Interquartile range
